# Supplementary material for: Identification of Freezing-Responsive microRNAs and Their Targets in Chinese Jujube by Small RNA and Degradome Sequencing
Source: Int J Mol Sci. 2026 Sep 4;27(17):7912. doi: 10.3390/ijms27177912 (PMC13566890; doi:10.3390/ijms27177912)
Supplement: Supplementary file 1 [file ijms-27-07912-s001.zip › Supplementary Material S2/novel_miR_38.pdf]

Provisional ID	:	novel_miR_38
Score total	:	1262.9
Score for star read(s)	:	3.9
Score for read counts	:	1260.9
Score for mfe	:	0.3
Score for randfold	:	-2.2
Score for cons. seed	:	
Total read count	:	2485
Mature read count	:	2483
Loop read count	:	0
Star read count	:	2

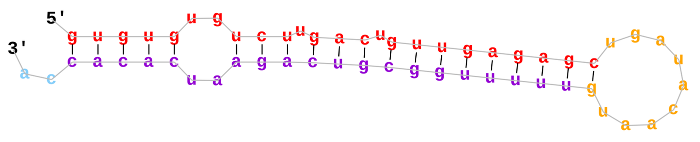

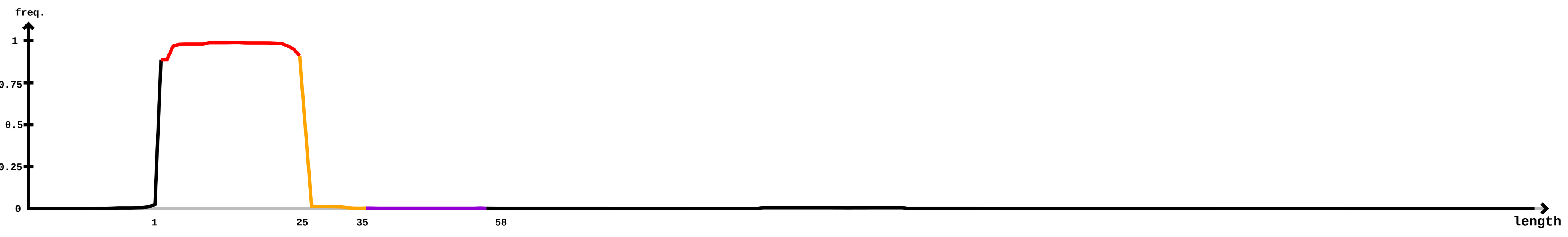

Mature Star

```
5'- ucuacugcccagugagaauugugugugucuugacuguugagagcugauacaauguuuuuggcgucagaaucacaccauauuuuuugccagccuuccucuugggcuaaaauuaauccuugcaggacucccacagcuucagcuagcucagggauaucugucugggugcgaauagacuuuauuuuuauaacuuugcccucauagucccuugccaccaguccaaugaagcuaacuccauuuuuaacugcugcau  -3'  obs
    ucuacugcccagugagaauugugugugucuugacuguugagagcugauacaaugouuuuggcgucagaaucacaccauauuuuuugccagccuuccucuugggcuaaaauuaauccuugcaggacucccacagcuucagcuagcucagggauaucugucugggugcgaauagacuuuauuuuuauaacuuugcccucauagucccuugccaccaguccaaugaagcuaacuccauuuuuaacugcugcau       exp
    .....(((.(((((((((.(((((..(((.(((.((((((((.........))))))))))))))..)))))...))))))....(((((.......)))))............(((((..((((..((((.....))))..))))..)))))((((..((((...((((......................))))..))))..))))...((((.((....)).))))....))))..))).  reads  mm  sample
    ........ccagugagaauugugugugugc.................  1  0  LT-1
```

sequence	reads	mm	sample
........ccagugagaauugugugugugc........	1	0	LT-1
.........cagugagaauugugGgugu........	1	1	JCK-1
..........agugagaauugugugugU........	2	1	LT-2
............ugagaauugugugugucuugac........	2	0	LT-2
.............gagaauugugugugucuugac........	3	0	LT-1
................aCuugugugugucuugacu........	1	1	LT-3
................aauugugugugucuugacuguuU........	2	1	JCK-2
.................auugugugugucuugacuguu........	1	0	JCK-2
.................auugugugugucuugacuguugag........	1	0	LT-1
..................uugugugugucuugacuguug........	1	0	LT-3
..................uugugugugucuugacuguugag........	1	0	LT-3
..................uugugugugucuugacuguugaga........	5	0	JCK-3
..................uugugugugucuugacuguugaga........	2	0	JCK-2
..................uugugugugucuugacuguugaga........	3	0	JCK-1
...................ugugugugucuugacuguuga........	1	0	LCK-3
...................ugugugugucuugacuguuga........	1	0	LT-1
...................ugugugugucuugacuguugag........	4	0	LCK-3
...................ugugugugucuugacuguuUag........	1	1	JCK-1
...................ugugugugucuugacuguuUag........	3	1	JCK-3
...................ugugugugucuugacuguugag........	1	0	JCK-3
...................ugugugugucuugacuguugag........	1	0	JCK-2
...................ugugugugucuugacuguugag........	3	0	JCK-1
...................ugugugugucuugacuguugaga........	3	0	LCK-3
...................ugugugugucuugacuguugagag........	1	0	JCK-1
...................ugugugugucuugacuguugagag........	2	0	JCK-2
...................ugugugugucuugacuguugagag........	2	0	JCK-3
...................ugugugugucuugacuguugagag........	2	0	LT-3
...................ugugugugucuugacuguugagag........	4	0	LT-1
...................ugugugugucuugacuguuUagag........	2	1	JCK-3
...................ugugugugucuugacuguugaAag........	1	1	LT-1
...................ugugugugucuugacuguuUagagc........	1	1	JCK-3
...................ugugugugucuugacuguugagagc........	1	0	JCK-1
...................Cgugugugucuugacuguugagagc........	1	1	LT-3

Mature Star

```
ucuacugcccagugagaauugugugugucuugacuguugagagcugauacaauguuuuuggcgucagaaucacaccauauuuuuugccagccuuccucuugggcuaaaauuaaauccuugcaggacucccacagcuucagcuagcucagggauaucugucugggugcgaauagacuuuauuuuuauaacuuugcccucauagucccuugccaccaguccaaugaagcuaacuccauuuuuaacugcugcau
...................ugugugugucuugacuguugagagc......   1     0   JCK-2
....................gugugugucuugacuguuga.........   1     0   LT-1
....................gugugugucuugacuguuUag........   3     1   JCK-2
....................gugugugucuugacuguugag........   6     0   LCK-3
....................gugugugucuugacuguugag........   3     0   LT-3
....................gugugugucuugacuguugag........   2     0   LT-2
....................gugugugucuugacuguuUag........   1     1   JCK-1
....................gugugugucuugacuguugag........   2     0   JCK-1
....................gugugugucuugacuguuUaga.......   2     1   LT-1
....................gugugugucuugacuguugaga.......   3     0   LT-1
....................gugugugucuugacuguugaga.......   12    0   LCK-2
....................gugugugucuugacuguugaga.......   14    0   LCK-1
....................gugugugucuugacuguugaga.......   2     0   JCK-3
....................gugugugucuugacuguuUagag......   1     1   JCK-1
....................gugugugucuugacuguugagag......   2     0   LCK-3
....................gugugugucuugacuguuUagag......   1     1   LT-3
....................gugugugucuugacuguugagag......   1     0   LT-3
....................gugugugucuugacuguuUagag......   3     1   LT-2
....................gugugugucuugacuguuUagag......   1     1   JCK-3
....................gugugugucuugacuguugaAag......   1     1   LT-3
....................gugugugucuugacuguugagag......   2     0   LT-1
....................gugugugucuugacuguugagagc.....   283   0   LT-1
....................gugugugucuugaAuguugagagc.....   1     1   LCK-3
....................gugugugucuugacuguugagagc.....   133   0   JCK-1
....................Augugugucuugacuguugagagc.....   1     1   LT-1
....................Augugugucuugacuguugagagc.....   1     1   LCK-3
....................gugugugucuugacuguugagagc.....   124   0   JT-1
....................gugugugucuugacuguuUagagc.....   12    1   LT-2
....................gugugugucuugacuguugagagc.....   28    0   JT-2
....................Augugugucuugacuguugagagc.....   3     1   JCK-2
....................gugugugucuugacuguugagagc.....   208   0   JCK-2
....................gugugugucuugacuguuUagagc.....   4     1   JT-2
....................gugugugucuugacuguugagCgc.....   1     1   LT-2
....................gugugugucuugacuguuUagagc.....   38    1   JT-1
....................gugugugucuugaAuguugagagc.....   3     1   LT-2
....................gugugugucuugCcuguugagagc.....   1     1   JCK-1
....................gugugugucuugacuguugagagU.....   1     1   LT-3
....................gugugugucuugacuguugagagc.....   58    0   LCK-2
....................gugugugucuugacuguugCgagc.....   1     1   JCK-2
....................gugugugucuugaAuguugagagc.....   1     1   LT-3
....................gugugugucuugacuguuUagagc.....   5     1   LCK-3
....................gugugugucuugacuguugaAagc.....   16    1   LCK-3
....................guAugugucuugacuguugagagc.....   1     1   LCK-3
....................gugugugucuugacuguuUagagc.....   8     1   JT-3
....................gugugugucuugacuguugNgagc.....   1     1   JCK-1
....................gugugugucuugacuguugaAagc.....   15    1   LT-1
....................gugugugucuugacuguugagagc.....   136   0   JCK-3
....................gugugugucuugacuguuUagagc.....   147   1   JCK-2
....................gugugugucuugacuguuUagagc.....   71    1   JCK-1
....................gugugugucuugacuguugagagc.....   34    0   JT-3
....................Uugugugucuugacuguugagagc.....   1     1   LCK-1
....................gugugugucuugacuguugagagc.....   269   0   LT-3
....................gugugugucuugacuguuUagagc.....   13    1   LT-1
....................gugugugucuugacuguuUagagc.....   16    1   LT-3
....................gugugugucuugacuguCgagagc.....   1     1   LT-3
....................Uugugugucuugacuguugagagc.....   1     1   LT-1
....................gugGgugucuugacuguugagagc.....   2     1   LT-3
....................gugugugucuugacuguugUgagc.....   1     1   JCK-2
....................gugugugucuugacuguugagagc.....   146   0   LT-2
....................gugugugucuugacuguugagagc.....   53    0   LCK-1
....................gugugugucuugacuguugagagc.....   177   0   LCK-3
....................gugugugucuugacuguugaAagc.....   9     1   LT-2
....................gugugugucuugacuguugagagU.....   1     1   LT-2
....................Augugugucuugacuguugagagc.....   1     1   LT-2
....................gugugugucuugacuguuUagagc.....   95    1   JCK-3
....................gugugugucuugacuguugaAagc.....   12    1   LT-3
.....................ugugugucuugacuguugagagc.....   1     0   JCK-2
......................gugugucuugacuguuga.........   1     0   LCK-3
......................gugugucuugacuguuUag........   3     1   JCK-2
......................gugugucuugacuguugaga.......   1     0   LT-3
```

```
          Mature               Star
ucuacugcccagugagaauugugugugucuugacuguugagagcugauacaauguuuuuggcgucagaaucacaccauauuuuuugccagccuuccucuugggcuaaaauuaauccuugcaggacucccacagcuucagcuagcucagggauaucugucugggugcgaauagacuuuauuuuuauaacuuugcccucauagucccuugccaccaguccaaugaagcuaacuccauuuuuaacugcugcau
......................gugugucuugacuguugaga................................................................................................................................................................................................  2   0   JCK-2
......................gugugucuugacuguugagag...............................................................................................................................................................................................  1   0   LT-1
......................gugugucuugacuguugagag...............................................................................................................................................................................................  39  0   LCK-1
......................gugugucuugacugGugagag...............................................................................................................................................................................................  1   1   LCK-1
......................gugugucuugacuguugagag...............................................................................................................................................................................................  31  0   LCK-2
......................Cugugucuugacuguugagag...............................................................................................................................................................................................  1   1   LT-3
......................gugugucuugacuguugagagc..............................................................................................................................................................................................  11  0   LT-3
......................gugugucuugacuguugagagc..............................................................................................................................................................................................  11  0   JCK-1
......................gugugucuugacuguugagagc..............................................................................................................................................................................................  6   0   LT-2
......................gugugucuugacuguuUagagc..............................................................................................................................................................................................  5   1   JCK-3
......................gugugucuugacuguugagagc..............................................................................................................................................................................................  19  0   JCK-2
......................gGgugucuugacuguugagagc..............................................................................................................................................................................................  1   1   LT-1
......................gugugucuugacuguugagagc..............................................................................................................................................................................................  4   0   LCK-3
......................gugugucuugacuguugagagc..............................................................................................................................................................................................  14  0   LT-1
......................gugugucuugacuguuUagagc..............................................................................................................................................................................................  1   1   LT-1
......................gugugucuugacuguuUagagc..............................................................................................................................................................................................  5   1   JCK-1
......................gugugucuugacuguugagagc..............................................................................................................................................................................................  17  0   JCK-3
......................gGgugucuugacuguugagagc..............................................................................................................................................................................................  1   1   LCK-3
......................gugugucuugacuguugaAagc..............................................................................................................................................................................................  5   1   LCK-3
......................gugugucuugacuguuUagagc..............................................................................................................................................................................................  7   1   JCK-2
......................gugugucuugacuguugaAagcu.............................................................................................................................................................................................  1   1   LT-1
......................gugugucuugacuguuUagagcu.............................................................................................................................................................................................  1   1   JCK-1
......................gugugucuugacuguugagagcu.............................................................................................................................................................................................  5   0   LT-2
......................gugugucuugacuguugagagcu.............................................................................................................................................................................................  2   0   LT-3
......................gugugucuugacuguuUagagcu.............................................................................................................................................................................................  1   1   JCK-3
......................gugugucuugacuguugaAagcu.............................................................................................................................................................................................  1   1   LT-3
......................gugugucuugacuguugagagcug............................................................................................................................................................................................  1   0   LT-3
......................gugugucuugacuguuUagagcug............................................................................................................................................................................................  2   1   JCK-2
......................gugugucuugacuguuUagagcug............................................................................................................................................................................................  1   1   JCK-1
......................gugugucuugacuguugagagcug............................................................................................................................................................................................  3   0   LT-1
.......................ugugucuugacuguugagagc..............................................................................................................................................................................................  2   0   LT-2
.......................ugugucuugacuguugagagc..............................................................................................................................................................................................  2   0   JCK-3
.......................ugugucuugacuguugagagc..............................................................................................................................................................................................  5   0   LT-3
.......................ugugucuugacuguugagagc..............................................................................................................................................................................................  2   0   LT-1
.......................ugugucuugacuguugagagc..............................................................................................................................................................................................  8   0   JCK-2
.......................ugugucuugacuguugaAagc..............................................................................................................................................................................................  2   1   LT-1
.......................ugugucuugacuguugagagc..............................................................................................................................................................................................  2   0   JCK-1
.......................ugugucuugacuguugaAagc..............................................................................................................................................................................................  2   1   LT-2
.......................ugugucuugacuguugagagcu.............................................................................................................................................................................................  1   0   JCK-1
.......................ugugucuugacuguugagagcu.............................................................................................................................................................................................  1   0   LT-3
........................gugucuugacuguugaAag...............................................................................................................................................................................................  1   1   LT-3
........................gugucuugacuguugagagcug............................................................................................................................................................................................  1   0   LT-1
........................gugucuugacuguuUagagcugau..........................................................................................................................................................................................  1   1   JCK-3
............................cuugacuguugagagcugau..........................................................................................................................................................................................  1   0   JCK-2
............................cuugacuguugagagcugauac........................................................................................................................................................................................  2   0   LT-2
............................cuugCcuguugagagcugauaca.......................................................................................................................................................................................  1   1   LT-3
............................cuugacuguugagagcugauaca.......................................................................................................................................................................................  4   0   LT-1
............................cuugacuguugagagcugauaca.......................................................................................................................................................................................  2   0   LT-2
............................cuugacuguugagagcugauaca.......................................................................................................................................................................................  1   0   JCK-2
............................cuugacuguugagagcugauaca.......................................................................................................................................................................................  2   0   JCK-1
............................cuugacuguuUagagcugauaca.......................................................................................................................................................................................  3   1   JCK-3
............................cuugacuguuUagagcugauaca.......................................................................................................................................................................................  1   1   LT-3
............................cuugacuguugagagcugauacaC......................................................................................................................................................................................  1   1   LT-3
............................cuugacuguugagagcugauacaC......................................................................................................................................................................................  2   1   LT-1
............................cuugacuguugagagcugauacaC......................................................................................................................................................................................  2   1   LCK-3
.............................uugacuguugagagcugaua.........................................................................................................................................................................................  1   0   JCK-2
.............................uugacuguugagagcugauaca.......................................................................................................................................................................................  1   0   LT-3
.............................uugacuguuUagagcugauacaau.....................................................................................................................................................................................  1   1   LT-1
................................acuguuUagagcugauacaauguu..................................................................................................................................................................................  1   1   JCK-1
................................acuguuUagagcugauacaauguu..................................................................................................................................................................................  1   1   JCK-2
...................................................auguuuuuggcgucagaaucacG................................................................................................................................................................................  2   1   JCK-2
...................................................auguuuuuggcgucagaaucacG................................................................................................................................................................................  1   1   JCK-3
......................................................uuuuuggcgucagaaucacac...............................................................................................................................................................................  1   0   JCK-2
......................................................uuuuuggcgucagaaucacac...............................................................................................................................................................................  1   0   JCK-3
.........................................................................accauauuuuuugccaAccuuc.........................................................................................................................................................  1   1   JCK-3
.........................................................................accauauuuuuugccaAccuuc.........................................................................................................................................................  2   1   JCK-2
............................................................................................................auuaauccuugcaggacucccacC......................................................................................................................  1   1   LT-1
..............................................................................................................uaauccuugcaggacucccacagc....................................................................................................................  1   0   LT-3
.......................................................................................................................caggacucccacagcuuca................................................................................................................  1   0   JCK-2
........................................................................................................................aggacucccaUagcuucagcuagc..........................................................................................................  2   1   JCK-2
```

Mature Star

```
ucuacugcccagugagaauugugugugucuugacuguugagagcugauacaauguuuuuggcgucagaaucacaccauauuuuuugccagccuuccucuugggcuaaaauuaauccuugcaggacucccacagcuucagcuagcucagggauaucugucugggugcgaauagacuuuauuuuuauaacuuugcccucauagucccuugccaccaguccaaugaagcuaacuccauuuuuaacugcugcau
........................................................................................................................aggacucccacagcuucagcuagc..........................................................................................................   3   0   JCK-1
........................................................................................................................aggacucccacagcuucagcuagc..........................................................................................................   3   0   JCK-3
........................................................................................................................aggacucccacagcuucagcuagc..........................................................................................................   1   0   JCK-2
........................................................................................................................aggacucccaUagcuucagcuagc..........................................................................................................   1   1   JCK-1
.......................................................................................................................................ucagcuaAcucagggauaucu..............................................................................................   1   1   JCK-1
..........................................................................................................................................gcuagcucagggauaucuguU...........................................................................................   1   1   JCK-2
..........................................................................................................................................gcuagcucagggauaucuguU...........................................................................................   1   1   JCK-3
....................................................................................................................................................................................................cauagucccuugccaccaguU.................................   1   1   JCK-1
```
